# Thickness-Dependent Piezoelectric Property from Quasi-Two-Dimensional Zinc Oxide Nanosheets with Unit Cell Resolution

**DOI:** 10.34133/2021/1519340

**Published:** 2021-02-25

**Authors:** Corey Carlos, Yizhan Wang, Jingyu Wang, Jun Li, Xudong Wang

**Affiliations:** Department of Materials Science and Engineering, University of Wisconsin–Madison, Madison, Wisconsin 53706, USA

## Abstract

A quantitative understanding of the nanoscale piezoelectric property will unlock many application potentials of the electromechanical coupling phenomenon under quantum confinement. In this work, we present an atomic force microscopy- (AFM-) based approach to the quantification of the nanometer-scale piezoelectric property from single-crystalline zinc oxide nanosheets (NSs) with thicknesses ranging from 1 to 4 nm. By identifying the appropriate driving potential, we minimized the influences from electrostatic interactions and tip-sample coupling, and extrapolated the thickness-dependent piezoelectric coefficient (*d*_33_). By averaging the measured *d*_33_ from NSs with the same number of unit cells in thickness, an intriguing tri-unit-cell relationship was observed. From NSs with 3*n* unit cell thickness (*n* = 1, 2, 3), a bulk-like *d*_33_ at a value of ~9 pm/V was obtained, whereas NSs with other thickness showed a ~30% higher *d*_33_ of ~12 pm/V. Quantification of *d*_33_ as a function of ZnO unit cell numbers offers a new experimental discovery toward nanoscale piezoelectricity from nonlayered materials that are piezoelectric in bulk.

## 1. Introduction

Piezoelectricity is a physical, reversible, and linear property that arises in materials with noncentrosymmetric crystal structures. The piezoelectric effect is described as the electromechanical coupling of electric dipole polarization of a material under mechanical strain. Piezoelectric materials are quintessential building blocks of modern technology and can be found in sensing, health monitoring, energy harvesting, and actuating applications [1–6]. Moreover, recent synthesis advances in the controlled-growth of transition-metal dichalcogenides (TMDs), hexagonal boron nitride (h-BN), and layered van de Waals (vdW) nanomaterials have led to new piezoelectric phenomena, where otherwise nonpiezoelectric bulk materials exhibit piezoelectricity as a result of lattice asymmetries, due to reduced dimensionality. So integral are piezoelectric materials to daily life, considerable research has been put forth to understand and predict piezoelectric properties in a broad range of nanomaterials [7–10].

Nanoscale piezoelectricity is of keen interest in recent decades. Theoretical studies predicting an enhanced piezoelectric effect of nanomaterials with reduced dimensionality have been pivotal arguments for experimental work. As a model piezoelectric material, the accepted piezoelectric coefficient (*d*_33_) of bulk ZnO is frequently reported (Table S1) as 12.4 pm/V [11]. Recent work has shown that both the morphology and thickness of the ZnO structure under investigation play a role in the overall piezo-response [12–15]. Through density functional theory (DFT) calculation, Agrawal et al. showed a near 50-fold increase in the piezoelectric coefficient (*d*_33_) of subnanometer wurtzite ZnO-NWs (50.4 C/m^2^ for 0.6 nm NW) with respect to bulk ZnO (0.6 C/m^2^) [8]. The enhanced piezoelectric effect was attributed to surface atom restructuring where the volume density of surface atoms dominates at the nanoscale, resulting in a larger polarization per unit volume. In a later study, using a molecular dynamics (MD) model, Momeni et al. calculated the piezoelectric coefficient of ZnO nanobelts with thicknesses ranging between 1 and 4 nm. They too found a similar trend which predicted an increased *d*_33_ for decreasing thicknesses (1.639 to 2.322 C/m^2^ when the thickness decreased from 4 to 1 nm) [9]. Although the predicted piezo coefficients in the latter study were smaller than the former, the smaller *d*_33_ values could be attributed to the limited fidelity of surface atoms within the scope of a strict ion approximation in the MD method. Regardless, the computational work has been a point of intrigue when studying the nanoscale piezoelectric properties.

Finding a suitable piezoelectric material that is stable in the given size regime has been an experimental challenge. Two-dimensional ZnO nanosheet (NS) structures synthesized by ionic layer epitaxy (ILE) offer an ideal platform for studying the nanoscale piezoelectric properties. Our recent synthesis advances enabled growth of ZnO NSs with unit-cell level control of the thickness, providing a unique material system to investigate the thickness-related piezoelectric property at the nanometer scale [16–19]. In this work, by optimizing the tip-bias conditions, we quantified the nanoscale piezoelectric coefficient, *d*_33_ from wurtzite ZnO NSs within the thickness range from 1 to 4 nm. A tri-unit-cell dependency of *d*_33_ was observed, where a bulk-like *d*_33_ was obtained from NSs with 3*n* unit cell thickness (*n* = 1, 2, 3), and ~30% higher *d*_33_ was obtained from NSs with other thicknesses. This work provides an intriguing experimental observation of the nanoscale piezoelectricity with a unit-cell thickness resolution.

## 2. Experiments

### 2.1. Growth of ZnO Nanosheets

ZnO NSs were synthesized by ILE (Figure S1). The details of solution preparation and materials characterization have been reported extensively in previous works [16, 18, 19]. Briefly, a nutrient solution for ZnO growth was prepared using 50 mL, 25 mM Zn(NO_3_)_2_:hexamethylenetetramine precursor solution distrubuted in a 24 mL vial(s). An 8 *μ*L droplet of 1.8 mM sodium oleyl sulfate (SDS) in chloroform solution was evenly applied to the nutrient solution surface. The vials were then sealed and placed in a convection oven at 60°C for 20, 30, and 40 minutes. Modulating the time of reaction was used for achieving different thicknesses of ZnO NSs. After the vials were removed from the oven and cooled to room temperature, a Ti/Au (3 nm/17 nm)-coated Si-substrate was used to scoop the 2D ZnO NSs from the air/water interface and left to air dry. The Ti/Au-coated substrate served as the bottom electrode for piezoelectric force microscopy (PFM) measurements.

### 2.2. Piezoelectric Characterization

PFM measurements were performed in the contact regime using the dynamic contact electrostatic force microscopy (DC-EFM) mode on a Park Systems XE-70 multimode atomic force microscope (AFM) and Stanford Research 830 (SR830) lock-in amplifier (LiA). A MikroMasch NSC36/Pt cantilever with a tip radius of <30.0 nm and a nominal force constant of 2.0 N/m was chosen to prevent sample destruction. The ZnO NS sample, supported by Au/Si substrate, was affixed to a conductive AFM sample disk using silver (Ag) paste, ensuring a proper ground (~3.8–4.1 ohms) for PFM measurements. Preparation of the (0001) bulk ZnO (10 × 10 × 0.5 mm, University Wafer) was similar to the ZnO NSs. For the (0001) bulk, we deposited Ti/Au (10 nm/40 nm) on the top and bottom surfaces, after which the bottom was adhered to a conductive AFM-disk using Ag-paste.

## 3. Results and Discussion

**Figure 1:**
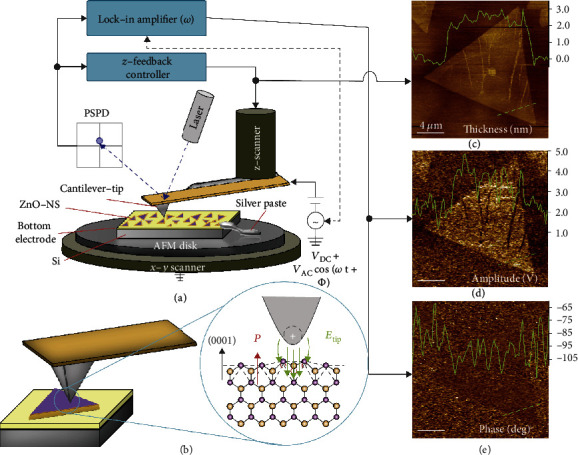
Characterization of the piezoelectric property on ZnO NSs. (a) Block diagram of the PFM setup. Both AC and DC voltages may be applied to a conductive cantilever-tip to induce mechanical deformations in a piezoelectric nanomaterial. The phase signal deviations from the driving frequency, as well as the physical deflections of the cantilever, establish the signals used for the contrast mechanisms needed to generate the topography (c), amplitude (d), and phase images (e). (b) Schematic showing raster scans a cantilever-tip driven at desired AC voltage inducing a local electric field. Inset: under an external electric field, the ZnO-nanosheets will exhibit mechanical displacement along the *c*-axis normal to the surface.

A block diagram schematic of the PFM measurement setup is shown in Figure 1(a). In this method, a conductive cantilever tip is electrically biased forming an electric field *E*_tip_ between the cantilever-tip and bottom electrode of the sample substrate as shown in Figure 1(b). During the measurement, the biased tip raster scans across the nanosheets and substrate surfaces. When there is an alternating current (AC) cantilever driving voltage *V*_C_ = *V*_0_ cos(*ωt* + *φ*), multiple interactions can be expected at the tip-sample interface. The predominant interaction between the AFM tip and a normal sample surface is the van der Waals interaction, which directly feedbacks to the local tip displacement or amplitude (*A*_z_) and measured by the AFM photodetector. Figure 1(c) shows a typical topography image of a ZnO NS when only *A*_z_ is recorded, which can typically give a vertical resolution of 0.03 nm by selecting an appropriate cantilever tip.

When the sample is piezoelectric with a finite piezoelectric response along the vertical direction (i.e., *d*_33_), additional vertical displacement can be expected as a result of the inverse piezoelectric effect (Δ*A*_z_ = *d*_33_ · *V*_c_) (as shown in Figure 1(b)). Ideally, the piezoelectric coefficient can be determined following the relation:

(1) $$\begin{array}{c} ∆A_{z}=d_{33}V_{0}\cos\left(\omega t+\varphi \right). \end{array}$$

The Δ*A*_z_ can therefore be recorded by the photodetector in terms of an additional voltage signal and plotted to show the real-time piezoelectric responses (Figure 1(d)). In addition, the quadrature phase components (*φ*) can provide additional information about local domain polarization *P* under the applied electric field. For *P* occurring completely parallel or antiparallel to the applied electric field *E*, the corresponding phases would be *φ* = 0° or *φ* = 180°, respectively (Figure 1(e)). Using this information, one can make observations about the domain polarization orientation. In ZnO, the lack of symmetry along the *c*-axis leads to a normal dipole moment between the Zn- and O-face, resulting in out-of-plane polarization. Stated plainly, if the orientation of the *c*-axis for the measured ZnO sample were to be completely parallel to the applied *E*-field, then one would expect a phase signal of 0° or 180°. Therefore, combining the topography, amplitude, and phase measurements allows full visualization of nanoscale piezoelectric phenomena using PFM.

However, electrostatic effects can contribute to, and sometimes dominate the amplitude response in PFM measurements, particularly when Δ*A*_z_ or *V*_C_ is too small [20, 21]. Many studies have shown methods for mitigating the electrostatic effects by selection of stiff cantilevers, strong indention forces, application of direct current (DC) voltages to the cantilever-tip, or combinations thereof [22, 23]. Due to the nanoscale nature of the ZnO NS, using stiff cantilevers or applying strong indentation forces cannot offer a solution as they increase the potential for damage during the scanning process. Additionally, the semiconducting properties of ZnO limits the application of DC voltages, as the likelihood of charge migration and interaction at the tip-sample interface would further contribute to electrostatic effects. Moreover, when *V*_C_ is too high, significantly stronger tip-sample coupling may be induced. This is a result of induced and accumulated surface charge under high electric field, leading to a capacitive coupling behavior at the tip-sample interface. It has been shown that this capacitive effect can be optimized between a bulk sample and cantilever-tip to obtain more accurate piezoelectric responses [24]. However, this approach does not suite materials in the nano-regime, because tip-sampling coupling cannot be definitively attributed to the nanometer thick sample. It is more likely that this capacitive coupling leads to extrinsic lattice constraints placed on the NS by the cantilever-tip and supporting substrate, which may introduce a higher level of noise to the measurement and suppress the actual piezoelectric responses of the NS. Therefore, in order to accurately realize the inverse piezoelectric response of a ZnO NS, an appropriate driving voltage regime must be evaluated and selected.

**Figure 2:**
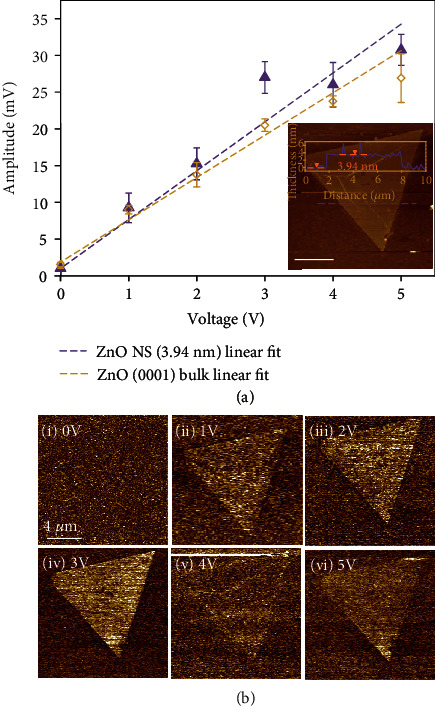
PFM driving voltage relationship. (a) Measured amplitude responses of single-crystal (0001)-oriented ZnO bulk crystal and a ZnO NS with a thickness of 3.94 nm. Inset is an AFM topography image of the ZnO NS. (b) A series of PFM amplitude maps acquired from the same ZnO NS under driving voltages from 0 to 5 V. The increased contrast indicates the monotonic increase of PFM responses following the driving voltage.

Initially, the driving voltage relationship of the PFM measurement was investigated on a ZnO NS and compared to a (0001) bulk ZnO crystal. The sample ZnO NS had a thickness of 3.94 nm (inset of Figure 2(a)). Experimental results for the PFM amplitudes (Δ*A*_Z_) as a function of *V*_0_ are shown in Figure 2(a). Corresponding amplitude images from the ZnO NS are given in Figure 2(b). When the driving voltage was zero, the PFM amplitude was not detectable from both the ZnO NS and bulk crystal samples. Particularly, the amplitude image contrast from the NS was completely blended with the substrate (Figures 2(b)–2(i)), suggesting that contributions from other artifacts were minimized. As *V*_0_ rose from 0 to 5 V, corresponding PFM amplitude increased almost linearly. For the bulk (0001) ZnO crystal, the PFM responses exhibited a good linearity with a slope of 5.76 × 10^−3^ and *R* value of 0.9942. A more obvious drop of amplitude could be observed at 5 V, which was possibly due to stronger electric fields generated at the tip-sample interface leading to charge migration and screening of the piezoelectric response. A similar trend was also obtained from the ZnO NS sample. Obviously, a higher amplitude was obtained from the ZnO NS surface as the driving voltage increased (Figure 2(b) ii–v), indicating higher vertical displacements due to the piezoelectric effect. Although a linear relationship could still be derived, values measured from ZnO NS exhibited higher fluctuation with *R* = 0.9876. The slope was determined to be 6.65 × 10^−3^, slightly higher than the bulk crystal.

The piezoelectric coefficient *d*_33_ was then calculated based on the PFM amplitude response, Δ*A*_Z_ under a given *V*_0_ following Equation (1). Our calculation of the amplitude response was based on the measured converse piezoelectric response of the sample, as detected by the AFM photodetector, which was then subtracted by an offset amplitude signal. An offset was used to account for the inherent system and environmental noise not filtered by the LiA. To measure the offset, the tip was raised ~100 nm above the surface of the sample to generate a mean value of the environmental noise. At this point, a PFM scan was performed, and an amplitude image was collected (Figure S2). The average amplitude signal response of the offset scan image was then subtracted from the average amplitude response directly measured from the ZnO NS surface to acquire Δ*A*_Z_.

**Figure 3:**
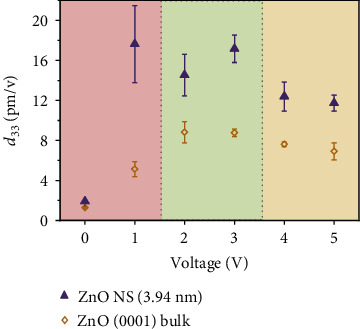
The calculated piezoelectric coefficients *d*_33_ for a bulk (0001) ZnO crystal (yellow diamonds) and a single ZnO NS (purple triangles) obtained under a series of driving voltages from 0 to 5 V.

As shown in Figure 3, the calculated *d*_33_ were generally smaller at *V*_0_ = 1 V with larger fluctuation for both ZnO bulk crystal and NS samples. The fluctuation was determined from the extracted *d*_33_ values of amplitude responses measured during the driving voltage scans shown in Figure 2(a). The large fluctuation could be attributed to the overwhelming electrostatic contributions between the tip and sample surface. As *V*_0_ increased to above 2 V, the *d*_33_ reached a relatively stable value of 8.83 pm/V for the bulk ZnO with a variation of ±12% (±1.06 pm/V). The *d*_33_ decreased slightly to 6.91 pm/V as *V*_0_ reached 5 V and a variation of ±12% (±0.85 pm/V) was recorded again. The overincreased *V*_0_ could give rise to an unwanted parasitic effect due to an AC-coupling between the tip-sample surface, which would suppress the amplitude response and induce signal fluctuation, resulting in lower *d*_33_ with higher variation. The same trend could be observed from the NS sample but with more obvious responses due to the extremely large surface-to-volume ratio. At low *V*_0_ (1V), the electrostatic interaction yielded a substantially large signal fluctuation from the ZnO NS (±20%) with a much higher *d*_33_ value (17.6 pm/V) compared to the bulk. This high value was also possibly exaggerated due to the electrostatic interaction. As *V*_0_ increased to 2 and 3 V, *d*_33_ from ZnO NS reduced to 14.5 pm/V (±14%) and 17.2 pm/V (±8%), respectively. Further increased *V*_0_ to 4 and 5 V yielded even lowered *d*_33_ of 12.4 pm/V (±12%) and 11.7 pm/V (±8%). Although the trend was similar, the absolute values of *d*_33_ were generally higher than those measured from the bulk sample, suggesting the effectivity of size confinement to enhanced piezoelectric responses.

Upon inspection, the driving voltages described above can be categorized into three regions. The electrostatic effects were most notable at low driving voltage region (*V*_0_ < 1.5 V), which led to large variations of amplitude response and *d*_33_. This region is marked in red in Figure 3. At higher voltages (*V*_0_ > 3.5 V, marked in yellow), the parasitic coupling between the sample and cantilever-tip becomes substantial, and a suppression of the PFM amplitude was induced and thus lowered *d*_33_. As such, a driving voltage of 2–3 V (green region) was found most reliable to represent the intrinsic piezoelectric response. In this region, the determined *d*_33_ (8.83 pm/V) from bulk ZnO crystal was also in good agreement with the reported values [11, 12]. *d*_33_ from the 3.94 nm ZnO NS was determined to be 14.5 pm/V.

**Figure 4:**
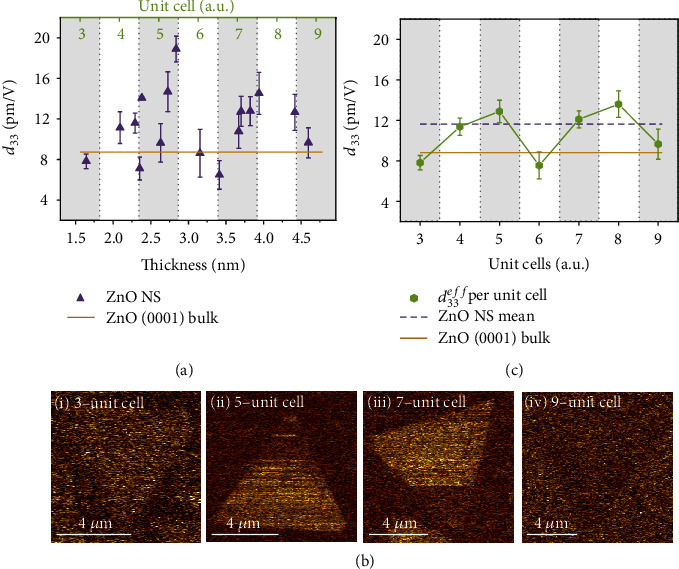
Thickness dependent piezoelectric property. (a) The piezoelectric coefficient *d*_33_ determined for ZnO NSs with various thicknesses measured using PFM at a tip bias of 2 V. Yellow line marks the bulk *d*_33_ value. (b) Representative PFM amplitude images obtained from ZnO NSs with thicknesses equivalent to 3, 5, 7, and 9 unit cells. (c) Effective piezoelectric coefficients as a function of number of unit cells calculated from NSs with equivalent unit cell thickness. Yellow line marks the bulk *d*_33_ value. Purple-dashed line marks the mean value of elevated *d*_33_ from NSs with thicknesses equivalent to 4, 5, 7, and 8.

After determining the appropriate *V*_0_ range, 2 V was used as the driving voltage to quantify the piezoelectric property of ZnO NSs in correlation to their thicknesses. By varying the growth time, ZnO NSs with thicknesses in the range of 1–4 nm were prepared in large quantity. Representative AFM topography images showed the well-controlled thickness variation (Figure S2). These nanosheets were examined by PFM and the extracted *d*_33_ values were plotted as a function of thicknesses in Figure 4(a), where the value determined from bulk ZnO (8.83 pm/V) was marked by the yellow line for reference. In general, a majority of the NSs (>75%) exhibited *d*_33_ greater than the bulk value within a relatively large range (6.48–18.9 pm/V). Most NS samples exhibited a relatively large deviation in their *d*_33_ values (~14%), although there was no apparent trend in the thickness of NSs and the deviation of the extracted *d*_33_, which was calculated (S1) after measuring four separate locations on the surface of each nanosheet (Figure S3). Corresponding PFM amplitude scan images of representative ZnO NSs with different thicknesses (one for each number of unit cell, see Discussion below) were shown in Figure 4(b). The contrast difference from triangular NSs could be clearly observed from these images, evidencing the different levels of piezoelectric responses.

It should be noted that in the nanometer scale, the NS thickness is not continuous and should be related to the number of unit cells along the thickness direction, i.e., the [0001] direction of ZnO [19]. Considering the lattice constant of wurtzite ZnO along the *z*-direction is 5.2 Å, the measured thicknesses were converted to the number of unit cells, providing a more realistic comparison of the thickness relationship. Assuming the standard uncertainty in topological measurement was uniform for all thickness samples, the number of unit cell (*n*) was correlated to the measured thickness (*d*) by the theoretical thickness (*n* · *d*_0_) within 50% difference of one unit cell height (*d*_0_), i.e., *d*<*n* · *d*_0_ ± 50% · *d*_0_. Thus, the effective piezoelectric coefficient (*d*_33_^eff^) as a function of number of unit cell thickness was determined by taking the average value of the *d*_33_ for ZnO-NS within the same *n*. These values were also plotted in Figure 4(c) as green dots. It is interesting to observe that NSs with thickness of 3, 6, 9 unit cells all had an average *d*_33_ value close to the bulk value (7.82 ± 0.7 pm/V, 7.55 ± 1.3 pm/V, and 9.66 ± 1.5 pm/V for 3, 6, and 9 unit cell NSs, respectively). In a similar method, we compared the phase response of the ZnO NSs to the (0001) ZnO bulk crystal that showed for increasing thicknesses, the phase response of the nanosheets trended toward the bulk behavior (Figure S4).

Nevertheless, NSs with the unit cell thicknesses that are not a multiple of 3, i.e., 4, 5, 7, and 8 unit cells, all exhibited a significantly higher *d*_33_ within a narrow range; 11.4 ± 0.9 pm/V, 12.9 ± 1.1 pm/V, 12.1 ± 0.9 pm/V, and 13.6 ± 1.3 pm/V, respectively. An average of all the nanosheets in this study is shown by the purple dashed line at 11.6 pm/V in Figure 4(c). This corresponds to 24% increase of the *d*_33_ compared to the bulk value of 8.83 pm/V shown by the yellow line. While theoretical work has predicted the piezoelectricity enhancement in the nanoscale wurtizite ZnO, which are mostly due to the surface effect, this tri-unit-cell relationship has not been discussed. This may not be the same reason as those suggested by the odd-layer-related piezoelectricity in layered materials as a result of lattice anisotropy [25]. However, at this point, we were not able to provide a quantitative explanation of how the triple unit cell configuration is related to the bulk-like piezoelectricity in the nanometer scale. This phenomenon may be resulted from underestimated surface-bulk lattice interaction in continuous 3D lattices, which needs to be further investigated by simulation approaches with more appropriately defined boundary conditions.

## 4. Conclusion

In summary, an experimental approach towards quantifying the piezoelectric property at the nanoscale was conducted using PFM on micrometer-sized ZnO NSs with well controlled thicknesses in the range of 1 to 4 nm. The effects of electrostatic forces and tip-sample interactions have been shown being able to introduce unignorable influences to the measured piezoelectric responses, especially where traditional methods applied in the so-called strong contact regime, would result in surface damage of nanometer thick ZnO. As a model piezoelectric, wurtzite ZnO was synthesized in a morphology of a free-standing NS with unit cell thickness control. The piezoelectric coefficient *d*_33_ was extrapolated from the PFM amplitude under selected driving voltage. By converting the AFM-measured thickness to number of unit cells, the averaged *d*_33_ exhibited an interesting relationship with the thickness. ZnO NSs with tri-unit-cell thicknesses exhibited a bulk-like value (~9 pm/V). A significantly higher *d*_33_ of ~12 pm/V was obtained from ZnO NSs with thicknesses that do not have 3*n* unit cell thickness. This experiment observation of thickness-dependent *d*_33_ suggests a different angle for investing the quantum-confined piezoelectricity from 2D lattices of nonlayered materials that are piezoelectric in bulk.

## Data Availability

The datasets generated and/or analyzed during the current study are available from the corresponding author upon reasonable request.

## Supplementary Materials

Supplementary Materials S1 Calculation of piezoelectric coefficient. Table S1: previously reported values for the piezoelectric coefficient (*d*_33_) of ZnO. Figure S1: scanning electron microscope (SEM) images of ZnO nanosheets synthesized via ionic layer epitaxy (ILE). SEM images show a good distribution of nanosheets that exhibit triangular morphology. Figure S2: (a) (i–vii) AFM topography images of ZnO nanosheet. (b) (i–vii) Thickness profile of nanosheets ranging between 1 and 4 nm, and (c) (i–vii) PFM amplitude response images of measured ZnO NSs corresponding to 3 to 9 unit cell. Figure S3. (a-i) Typical AFM topography image of ZnO NS and corresponding (a-ii) amplitude response image. The topography image illustrates the 4 regions where the amplitude response was collected. (b-i) Topography of the reduced scan areas and corresponding (b-ii) amplitude response. (c) (i–iv) The extracted amplitude response of each region 1–4, (c-iv) offset scan image, and (c-vii) offset amplitude response image, where no topography is recorded since the tip is not in contact with the sample surface. Figure S4. (a) The measured phase response for each ZnO NS when compared to the measured ZnO (0001) Bulk crystal shows no apparent thickness related trend. (b) However, when taking the average phase response per unit cell, the phase response illustrates a tendency toward bulklike behavior as the NS thickness increases.
