# Supplementary material for: Thickness-Dependent Piezoelectric Property from Quasi-Two-Dimensional Zinc Oxide Nanosheets with Unit Cell Resolution
Source: Research (Wash D C). 2021 Feb 25;2021:1519340. doi: 10.34133/2021/1519340 (PMC7936626; doi:10.34133/2021/1519340)
Supplement: Supplementary Materials — S1 Calculation of piezoelectric coefficient. Table S1: previously reported values for the piezoelectric coefficient (d33) of ZnO. Figure S1: scanning electron microscope (SEM) images of ZnO nanosheets synthesized via ionic layer epitaxy (ILE). SEM images show a good distribution of nanosheets that exhibit triangular morphology. Figure S2: (a) (i–vii) AFM topography images of ZnO nanosheet. (b) (i–vii) Thickness profile of nanosheets ranging between 1 and 4 nm, and (c) (i–vii) PFM amplitude response images of measured ZnO NSs corresponding to 3 to 9 unit cell. Figure S3. (a-i) Typical AFM topography image of ZnO NS and corresponding (a-ii) amplitude response image. The topography image illustrates the 4 regions where the amplitude response was collected. (b-i) Topography of the reduced scan areas and corresponding (b-ii) amplitude response. (c) (i–iv) The extracted amplitude response of each region 1–4, (c-iv) offset scan image, and (c-vii) offset amplitude response image, where no topography is recorded since the tip is not in contact with the sample surface. Figure S4. (a) The measured phase response for each ZnO NS when compared to the measured ZnO (0001) Bulk crystal shows no apparent thickness related trend. (b) However, when taking the average phase response per unit cell, the phase response illustrates a tendency toward bulklike behavior as the NS thickness increases. [file 1519340.f1.pdf]

Supporting Information

Thickness-Dependent Piezoelectric Property from Quasi-Two-Dimensional Zinc Oxide Nanosheets with Unit Cell Resolution

Corey Carlos,[1] Yizhan Wang, [1] Jingyu Wang,[1] Jun Li,[1] Xudong Wang[*,1]

1. Department of Materials Science and Engineering, University of Wisconsin - Madison, Madison, Wisconsin 53706, United States

Email: XW: xudong.wang@wisc.edu

S1. Calculation of piezoelectric coefficient.

d_{33} = (PFM amplitude [V] – PFM amplitude offset [V]) ÷ lock in amp. gain [V/V] ÷ ratio

A-B (AC) gain to A-B (DC) gain [V/V] × A-B sensitivity [um/V] ÷ 5 ÷ ac bias amplitude [V] (1)

where PFM amplitude represents the PFM amplitude signal of sample, PFM amplitude

offset represents the PFM amplitude signal of the cantilever in free-space, lock in amp. gain = 1, ratio A-B (AC) gain to A-B (DC) gain = 1, A-B sensitivity = 0.0095 um/V and ac bias

amplitude = 2.000 V.

Table S1. Previously reported values for the piezoelectric coefficient (d_{33}) of ZnO.

**ZnO Structure**	**d_{33} (pm/V)**	**Reference**
Nanosheets (NS)	6.48 – 18.9	This Work
Bulk thin film (>1000 nm)	12.4**	[11]
Ultra-thin film (<10 nm)	23.7	[14]
Nanobelts (NB)	14.3 – 26.7	[12]
Nanorods (NR)	0.9 – 9.5	[13]
Hexagonal Nanoplatelet	18.9 – 22.5	[15]

**Typically quoted as the standard piezo coefficient (d_{33}) value obtained for bulk ZnO using piezo force microscopy (PFM). Reference numbers are referred to the main text.

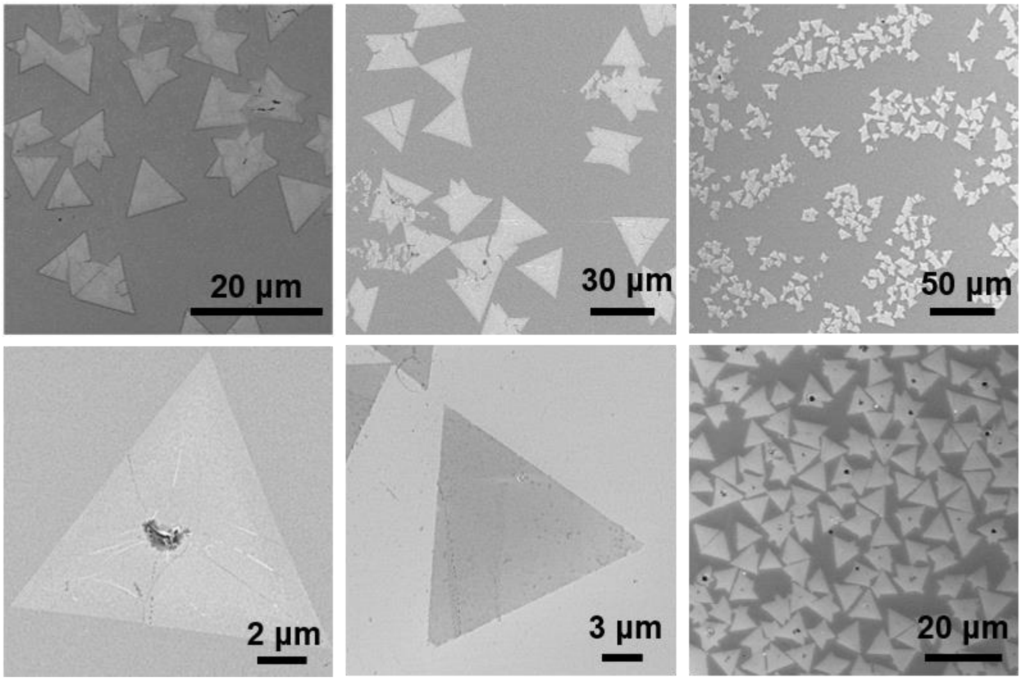

Figure S1. Scanning electron microscope (SEM) images of ZnO nanosheets synthesized via ionic layer epitaxy (ILE). SEM images show a good distribution of nanosheets that exhibit triangular morphology.

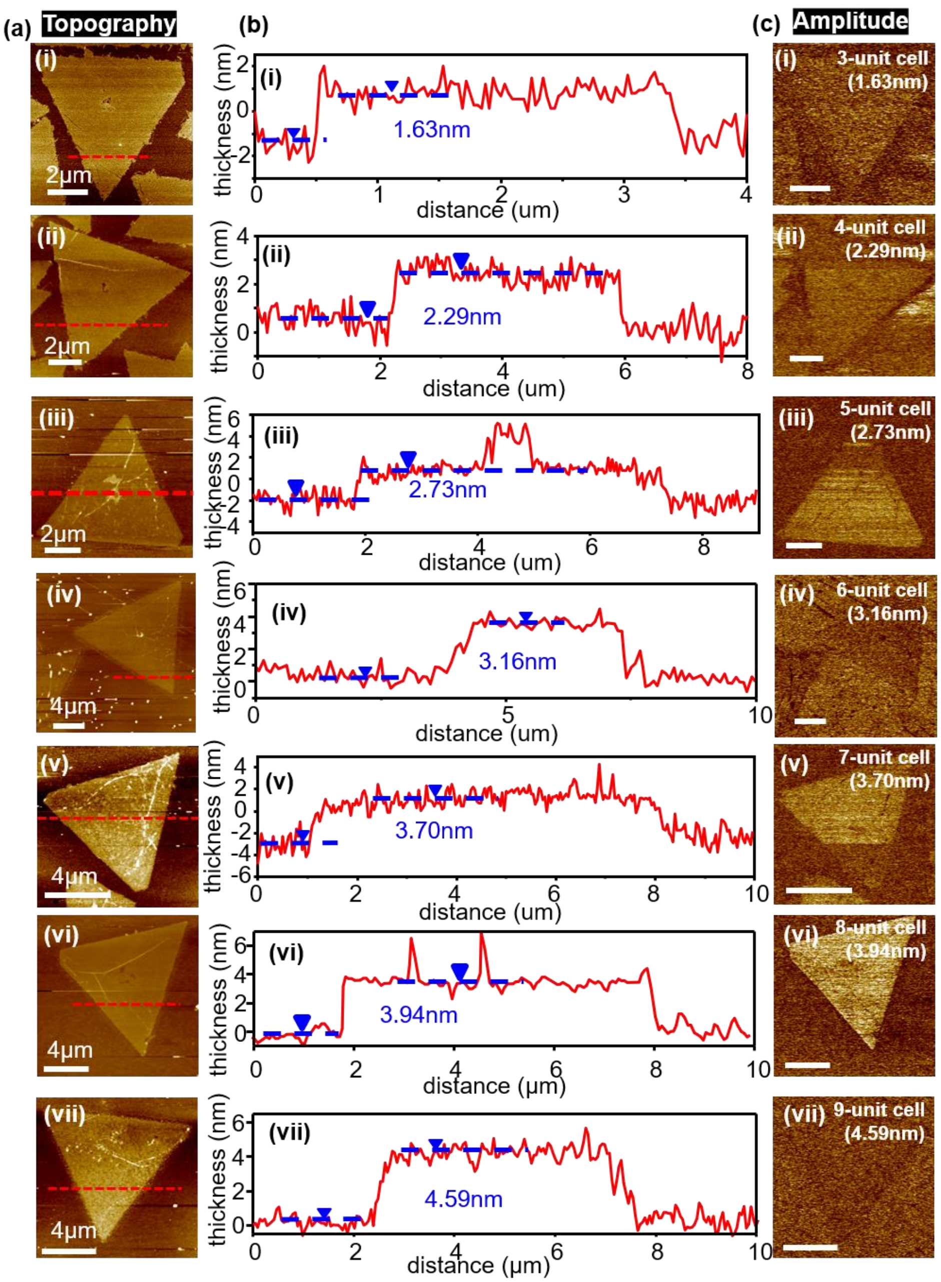

Figure S2. (a) [i-vii] AFM topography images of ZnO nanosheet. (b) [i-vii] Thickness profile of nanosheets ranging between 1 to 4 nm, and (c) [i-vii] PFM amplitude response images of measured ZnO NSs corresponding to 3 to 9 unit cell.

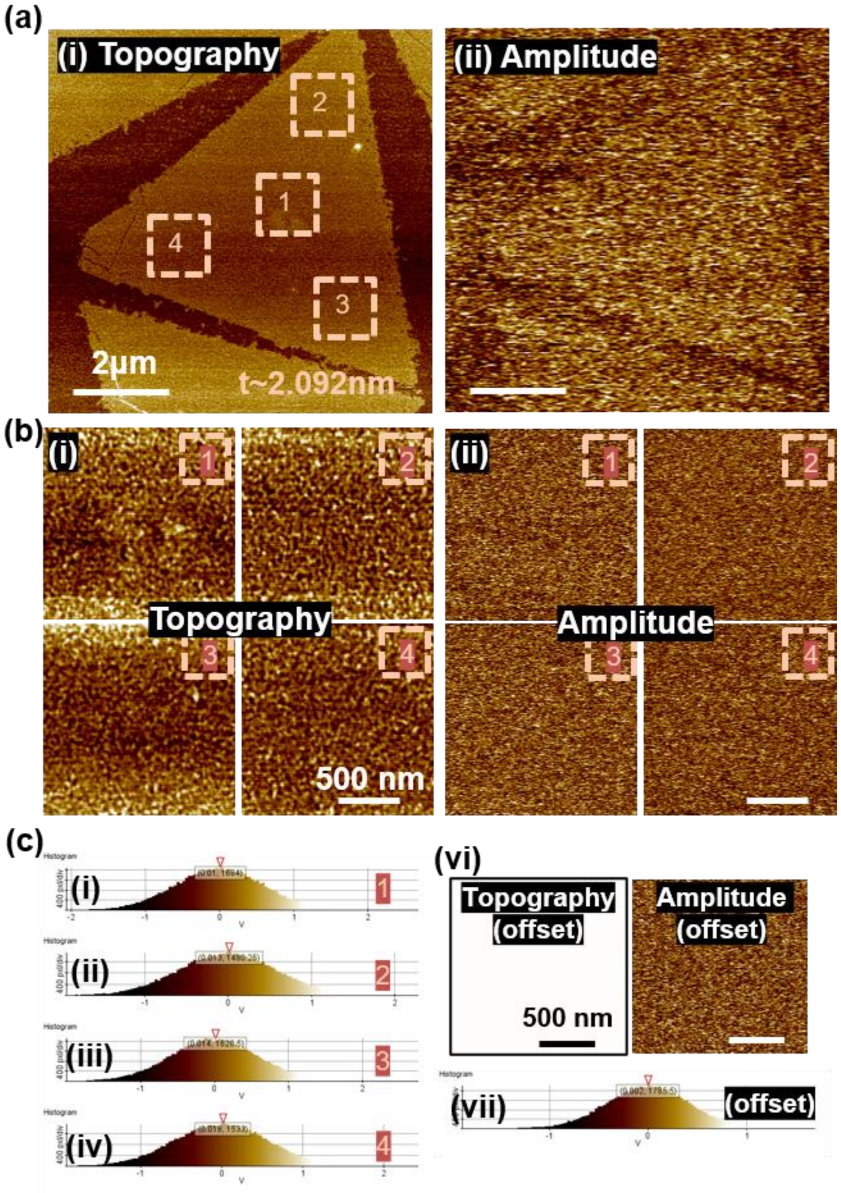

Figure S3. (a-i) Typical AFM topography image of ZnO NS and corresponding (a-ii) amplitude response image. The topography image illustrates the 4-regions where the amplitude response was collected. (b-i) Topography of the reduced scan areas and corresponding (b-ii) amplitude response. (c) [i-iv] The extracted amplitude response of each region 1 – 4, (c-iv) offset scan image, and (c-vii) offset amplitude response image, where no topography is recorded since the tip is not in contact with the sample surface.

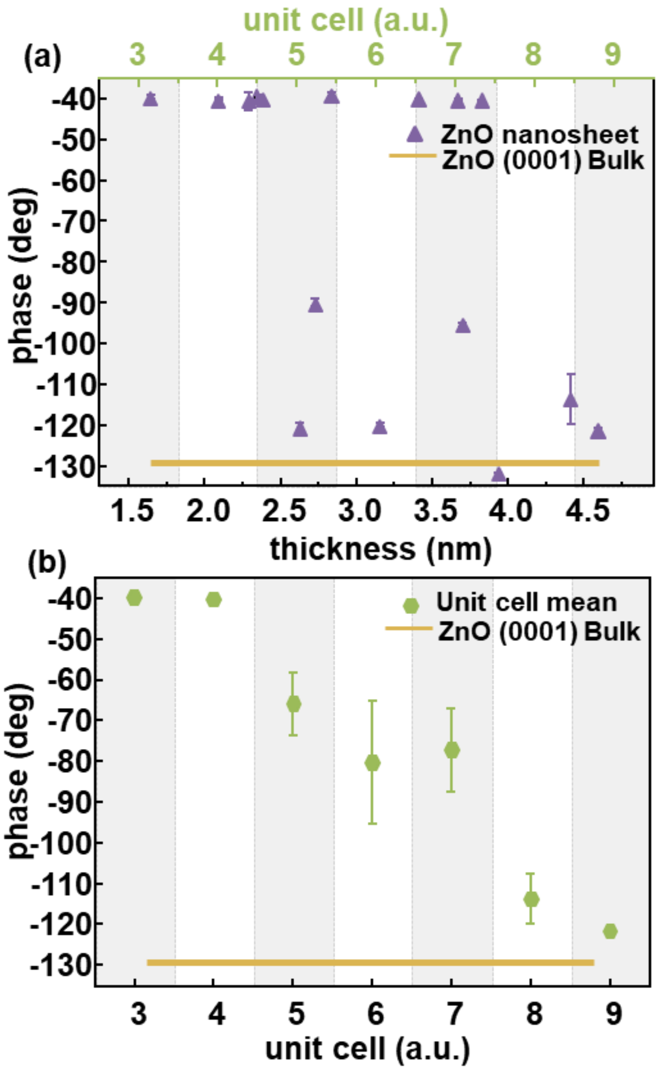

Figure S4. (a) The measured phase response for each ZnO NS when compared to the measured ZnO (0001) Bulk crystal shows no apparent thickness related trend. (b) However, when taking the average phase response per unit cell, the phase response illustrates a tendency toward bulk-like behavior as the NS thickness increases.
